# Suppressing Dengue-2 Infection by Chemical Inhibition of *Aedes aegypti* Host Factors

**DOI:** 10.1371/journal.pntd.0003084

**Published:** 2014-08-07

**Authors:** Seokyoung Kang, Alicia R. Shields, Natapong Jupatanakul, George Dimopoulos

**Affiliations:** W. Harry Feinstone Department of Molecular Microbiology and Immunology, Bloomberg School of Public Health, Johns Hopkins University, Baltimore, Maryland, United States of America; Colorado State University, United States of America

## Abstract

Dengue virus host factors (DENV HFs) that are essential for the completion of the infection cycle in the mosquito vector and vertebrate host represent potent targets for transmission blocking. Here we investigated whether known mammalian DENV HF inhibitors could influence virus infection in the arthropod vector *A. aegypti*. We evaluated the potency of bafilomycin (BAF; inhibitor of vacuolar H+-ATPase (vATPase)), mycophenolic acid (MPA; inhibitor of inosine-5′-monophosphate dehydrogenase (IMPDH)), castanospermine (CAS; inhibitor of glucosidase), and deoxynojirimycin (DNJ; inhibitor of glucosidase) in blocking DENV infection of the mosquito midgut, using various treatment methods that included direct injection, ingestion by sugar feeding or blood feeding, and silencing of target genes by RNA interference (RNAi). Injection of BAF (5 µM) and MPA (25 µM) prior to feeding on virus-infected blood inhibited DENV titers in the midgut at 7 days post-infection by 56% and 60%, and in the salivary gland at 14 days post-infection by 90% and 83%, respectively, while treatment of mosquitoes with CAS or DNJ did not affect susceptibility to the virus. Ingestion of BAF and MPA through a sugar meal or together with an infectious blood meal also resulted in various degrees of virus inhibition. RNAi-mediated silencing of several vATPase subunit genes and the IMPDH gene resulted in a reduced DENV infection, thereby indicating that BAF- and MPA-mediated virus inhibition in adult mosquitoes most likely occurred through the inhibition of these DENV HFs. The route and timing of BAF and MPA administration was essential, and treatment after exposure to the virus diminished the antiviral effect of these compounds. Here we provide proof-of-principle that chemical inhibition or RNAi-mediated depletion of the DENV HFs vATPase and IMPDH can be used to suppress DENV infection of adult *A. aegypti* mosquitoes, which may translate to a reduction in DENV transmission.

## Author Summary

Arboviruses utilize homologous host factors of the mammalian and insect cellular machinery to complete the infection cycle. Studies in both mammalian and insect cell lines have shown that virus infection can be suppressed through inhibition of host factors by chemical compounds that therefore could be developed into transmission blocking agents. However, similar studies have not been conducted in adult mosquitoes. Here we investigated the effect of four chemical compounds (bafilomycin, mycophenolic acid, castanospermine, and deoxynojirimycin), known to inhibit the host factors vacuolar H+-ATPase (vATPase), inosine-5′-monophosphate dehydrogenase (IMPDH) and glucosidases, on dengue virus replication in adult mosquitoes. We found that bafilomycin and mycophenolic acid suppressed dengue virus replication in adult mosquito guts when they were injected prior to dengue virus infection; however, castanospermine and deoxynojirimycin did not. Ingestion of bafilomycin and mycophenolic acid also inhibited virus replication. We showed that the predicted target genes of bafilomycin and mycophenolic acid function as virus host factors in adult mosquitoes through RNAi-mediated gene silencing. Inhibition of vATPase also decreases mosquito longevity and fecundity, thereby further compromising vector capacity. Our study demonstrated that chemical compounds or double stranded RNAs (dsRNA) can be used to suppress virus infection through inhibition of host factors in adult mosquitoes, thereby rendering such approaches interesting for the development of novel transmission-blocking strategies.

## Introduction

From a global health perspective, dengue virus (DENV) is currently the most important arbovirus transmitted by mosquitoes. Approximately 3.6 billion people are at risk of DENV infection, and 100 million people are infected annually [1]. Given the lack of registered antivirals or vaccines against DENV, a major effort to reduce DENV transmission has been concentrated on mosquito vector control. Although suppression of mosquito populations represents the most widely used dengue control strategy, this approach is hampered by insecticide resistance and the rapid adaptation and expansion of mosquitoes to urban areas [2]. Thus, the development of novel methods to reduce DENV transmission is urgently needed. Here, we investigated a novel transmission-blocking method that targets mosquito proteins (HFs) used by DENV for viral replication and transmission instead of directly targeting the mosquito or DENV for destruction.

DENV incubates in a mosquito for about 14 days before the mosquito is able to transmit the virus to a human host. The virus is ingested by the mosquito through infected blood, from which it infects the insect's midgut epithelial cells. There the virus replicates and then disseminates throughout the mosquito, including the salivary glands, where it further replicates and is then transmitted to a new human host [3]. During this extrinsic incubation period, the mosquito mounts an immune response against the virus that results in suppression of infection to various degrees. Previous studies have shown that the Toll, Janus kinase/signal transducer and activator of transcription (JAK/STAT), and RNA interference pathways control DENV restriction mechanisms in mosquitoes [4]–[6].

Several studies have identified mosquito genes that are essential for arbovirus replication and transmission and can therefore be considered DENV host factors (HFs) [7]–[10]. For example, mosquito prohibitin is a DENV HF that acts as a receptor protein to mediate DENV cell entry [7]. The mosquito and mammalian vacuolar H+-ATPase (vATPase) functions as a DENV HF by acidifying endosomes, a process that is important for viral fusion and the release of the viral genome into the cytoplasm [11], [12]. DENV also utilizes HFs that are involved in *de novo* pathways for RNA synthesis during viral replication [13]. Host glucosidase has also been shown to act as a DENV HF and is responsible for the proper folding and glycosylation of virus proteins [14], [15]. Other mosquito proteins have also been shown to act as virus agonists, but the mechanisms by which they influence virus infection remains unknown. For example, an *A. aegypti* cathepsin, an MD2-like protein, and NPC1-like factors have been shown to act as DENV agonists [16]. Thus, DENV HFs and agonists represent potential chemical- and vaccine-based transmission-blocking targets that could be developed into novel mosquito-based dengue control strategies.

In several studies, chemical compounds targeting DENV HFs in mammalian or insect cells have been shown to suppress DENV infection to various degrees [14], [15], [17]–[22]. However, the possible anti-dengue activities of such chemicals have not been studied in adult mosquitoes in order to assess their usefulness for transmission blocking. A chemical approach to inhibiting DENV HFs in the mosquito could circumvent the ecological impact of insecticide use and the probability of the virus's developing resistance to the blocking mechanism.

We investigated the ability of four putative DENV HF-inhibitor compounds, bafilomycin (BAF), mycophenolic acid (MPA), castanospermine (CAS), and deoxynojirimycin (DNJ), to block DENV infection of the mosquito midgut. We administered these compounds by various treatment methods, including injection and ingestion by sugar feeding or blood feeding. BAF is a well-characterized inhibitor of HFs for various viruses in mammalian and insect cell lines [18], [23]–[26]. It acts by interfering with vATPase function, thereby inhibiting the acidification of the endosome, a necessary step for viral entry [10], [27], [28]. MPA also exerts antiviral activity in mammalian and insect cells [13], [17], [29] by inhibiting the enzyme inosine 5′-monophosphate dehydrogenase (IMPDH), which is involved in the *de novo* pathway of guanosine nucleotide synthesis that is essential for viral RNA synthesis as well as host DNA and RNA synthesis [13]. CAS and DNJ are inhibitors of glucosidases that act as DENV HFs by ensuring the proper folding and glycosylation of viral proteins [14], [15].

## Materials and Methods

### Ethics statement

This study was carried out in strict accordance with the recommendations in the Guide for the Care and Use of Laboratory Animals of the National Institutes of Health. Mice were only used for mosquito rearing as a blood source according to approved protocol. The protocol was approved by the Animal Care and Use Committee of the Johns Hopkins University (Permit Number: M006H300). Commercial anonymous human blood was used for dengue virus infection assays in mosquitoes, and informed consent was therefore not applicable. The Johns Hopkins School of Public Health Ethics Committee has approved this protocol. Mosquito collections were performed in residences after owners/residents permission.

### Mosquito rearing and dissections, and cells cultures

Several *A. aegypti* strains (Rockefeller/UGAL [Rock], Singapore [SIN], and Puerto Triunfo [PTri]) [30] were used to test the function(s) of vATPase subunits by RNA interference (RNAi)-mediated gene silencing. The anti-DENV compounds assays and IMPDH gene silencing assay were performed with our standard lab strain, the Rock strain. The mosquitoes were maintained on a 10% sucrose solution at 27°C and 95% humidity with a 12-hr light/dark cycle [5]. Infected mosquitoes were dissected to collect the midguts at 7 days post-infection or the salivary glands at 14 days post-infection. Each mosquito body was dipped in 70% ethanol and rinsed twice in 1×PBS. The dissection was performed in 1 drop of 1× PBS, and the dissected midguts or salivary glands were transferred to a microcentrifuge tube containing 150 µl of MEM and stored at −80°C until used for virus titration. The C6/36 (*A. albopictus*) cell line that was used for DENV propagation was grown in minimal essential medium (MEM, Gibco) with 10% heat inactivated FBS, 1% L-glutamine, 1% penicillin-streptomycin, and 1% non-essential amino acids at 32°C with 5% CO_2_. The BHK-21 (baby hamster kidney) cell line that was used for plaque assays was maintained on Dulbecco's modified Eagle's medium (DMEM, Gibco) supplemented with 10% FBS, 1% L-glutamine, 1% penicillin-streptomycin, and 5 µg/ml plasmocin (Invitrogen) at 37°C and 5% CO_2_.

### DENV infection of mosquitoes

DENV serotype 2 (New Guinea C strain, DENV-2) was propagated in the C6/36 cell line. One milliliter of virus stock was used to infect a 75-cm^2^ flask of C6/36 cells at 80% confluence. Infection was allowed to proceed for 6 days, at which time the cells were harvested, centrifuged at 800 g for 10 min, and mixed 1∶1 with commercial human blood supplemented with 10% human serum and 1% 100 mM ATP (Thermo scientific). The infectious blood meal was maintained at 37°C for 30 min prior to feeding 5- to 7-day old mosquitoes.

### Injection of compounds

Bafilomycin (BAF) was obtained from Cayman Chemical, and mycophenolic acid (MPA), castanospermine (CAS), and deoxynojirimycin (DNJ) were obtained from Sigma Aldrich. Compounds were resuspended in 100% dimethyl sulfoxide (DMSO) and further diluted with PBS to yield a 10% DMSO in 1XPBS solution. Control mosquitoes were injected with the 10% DMSO in 1XPBS solution. Cold-anesthetized 6-day-old female mosquitoes were injected in the thorax and then transferred to paper cups and stored at 27°C overnight for next-day DENV infection.

### Ingestion of compounds via a sugar meal or blood meal

For ingestion of compounds through the sugar meal, various concentrations of BAF and MPA were mixed with a 10% sucrose solution to yield a 2.5% DMSO solution. Four-day-old females were fed on the solution for 2 days prior to, and every second day until the day of dissection after, ingestion of a DENV-infected blood meal. For ingestion of compounds through the DENV-infected blood meal, various concentrations of BAF and MPA were directly mixed with the blood to yield a 2.5% DMSO solution. Control groups were provided with either sucrose or blood meals containing an equal amount of DMSO.

### Gene silencing assays

The function of vATPase subunits and IMPDH as DENV HFs was assayed using RNAi-mediated gene silencing as described previously [31]. In brief, double-stranded RNAs targeting the following genes encoding various vATPase subunits were synthesized from PCR-amplified gene fragments using the HiScribe T7 *in vitro* transcription kit (NEBioLabs): vATPase subunit ac39 (vATP-ac39, AAEL011025), vATPase proteolipid subunit (vATP-V0B, AAEL012113), vATPase subunit f (vATP-f, AAEL002464), vATPase 16-kDa proteolipid subunit (vATP-16, AAEL000291) and IMPDH (AAEL009273), plus the GFP gene as a control. The primer sequences are listed in Table S1. Approximately 69 nl of dsRNAs (3 µg/µl; 200 ng/mosquito) in water was injected into the thorax of cold-anesthetized 4-day-old female mosquitoes using a nano-injector (Nanoject; Drummond Scientific) with a glass capillary. Three days after injection, mosquitoes were fed on a DENV-2-supplemented blood meal or were sacrificed for gene-silencing efficiency assays. After non-fed mosquitoes were removed, the blood-fed mosquitoes were maintained in the insectary under the conditions mentioned above for 7 days before midgut dissection or for 14 days before salivary gland dissection. Gene silencing was verified 3 days after dsRNA injection by real-time quantitative RT-PCR. Three independent biological replicate assays were performed for each gene, with the A. aegypti ribosomal *S7* gene as the internal control for normalization [32]. Mosquito samples were collected in RLT buffer (Qiagen) and then stored at −80°C until extraction. Total RNA was extracted from tissue samples using the RNeasy Mini Kit (Qiagen) according to the manufacturer's protocol. For cDNA synthesis, extracted RNA samples were treated with Turbo DNase (Ambion) at 37°C for 1 h before reverse transcription with a MMLV Reverse Transcriptase kit (Promega) according to the manufacturer's instructions. The cDNA was then used to determine gene expression by qPCR using the SYBR Green PCR Master Mix with gene-specific primers. The primers for each gene are presented in Table S2.

### DENV titration by plaque assay

DENV-NGC titers in midguts were determined by plaque assay on BHK-21 cells. Frozen midguts were thawed, homogenized in DMEM with a Bullet Blender (NextAdvance), serially diluted, and then inoculated onto cells seeded to 80% confluence in 24-well plates (100 µl per well). Plates were rocked for 15 min at room temperature, and then incubated for 45 min at 37°C and 5% CO_2_. Subsequently, 1 ml of DMEM containing 2% FBS and 0.8% methylcellulose were added to each well, and plates were incubated for 5 days at 37°C and 5% CO_2_. Plates were fixed with a methanol/acetone mixture (1∶1 volume) for at least 1 h at 4°C, and plaque-forming units were visualized by staining with 1% crystal violet solution for 10 min at room temperature.

### Longevity, fecundity, and egg hatchability assays

Longevity, fecundity, and egg hatchability assays were performed after vATPase and IMPDH were silenced with dsRNAs as described previously [33]. For the longevity assay, 20 four-day old Rock strain adult female mosquitoes that had been injected with dsRNAs targeting either GFP, vATPase (V0B), or IMPDH were kept in a wax-lined cardboard cup at 27°C with 70% humidity and maintained on a sterile 10% sucrose solution. Three biological replicates were performed, and all groups were monitored daily for survival until all mosquitoes had perished. The survival percentage represents the mean survival percentage for all three biological replicates as described previously [33]. For the fecundity assay, 30 four-day old GFP dsRNA-, vATPase (V0B) dsRNA-, or IMPDH dsRNA -injected mosquitoes were allowed to feed on human blood through an artificial membrane feeder for 30 min. The fed mosquitoes were transferred to individual 50 ml centrifuge tubes (one mosquito per tube) outfitted with moistened filter paper at the base of the tubes, and incubated under normal rearing conditions. Eggs oviposited on filter paper were counted after 2 days using light microscopy. Female mosquitoes that did not produce eggs on day 2 were maintained and re-examined on day 3. After each count, eggs were submerged in a standard larval pan for rearing according to standard methods. First- to second-instar larvae were counted to determine the larval hatch rate. The fecundity and larval hatch-rate assays were performed in three biological replicates, and the number of eggs laid by each female and the respective hatch rates were used to calculate mean values.

### Statistical analyses

The midgut or salivary gland DENV titers of the control groups and experimental groups were compared using the plaque assay results from at least three biological replicates. Mann-Whitney U-tests and Kruskal-Wallis tests with Dunn's multiple comparison test were used when appropriate. A 2-way ANOVA was performed to analyze the longevity assay data in addition to Kaplan–Meier survival analysis and Wilcoxon tests (Table S3). A Mann-Whitney U-test and a Student's t-test were used to calculate p-values and determine the significance of fecundity and fertility, respectively. Statistical analyses were conducted using the GraphPad Prism statistical software package (Prism 5.05; GraphPad Software, Inc.). Statistical significance is indicated with asterisks: *, p<0.05; **, p<0.01; ***, p<0.001. Descriptive statistics for DENV infection assays are presented in supplementary table S2.

### List of accession numbers/ID numbers for genes

vATPase subunit ac39 (vATP-ac39, AAEL011025), vATPase proteolipid subunit (vATP-V0B, AAEL012113), vATPase subunit f (vATP-f, AAEL002464), vATPase 16-kDa proteolipid subunit (vATP-16, AAEL000291) and IMPDH (AAEL009273).

## Results

### Injection of BAF and MPA prior to ingestion of DENV suppresses virus infection of the mosquito gut

**Figure 1:**
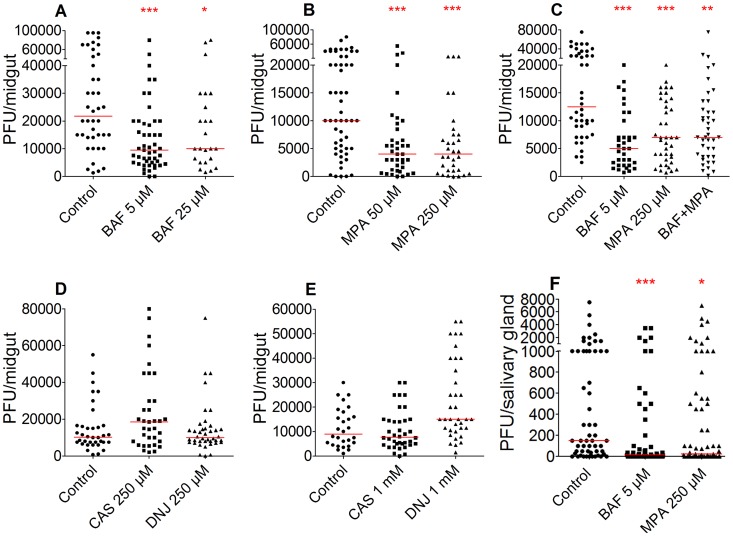
Injection of BAF or MPA suppresses DENV infection in mosquito midguts. A) DENV titers of bafilomycin (BAF; 5 µM and 25 µM)-injected mosquito midguts at 7 days post-blood meal (dpbm) were compared to those of the DMSO-injected control group. Each data point represents virus infection intensity (titer) from an individual midgut. Each mosquito was injected with 0.345 pmole (214.9 pg) or 1.725 pmole (1.1 ng) of BAF. B) DENV titers of mycophenolic acid (MPA; 50 µM and 250 µM)-injected mosquito midguts at 7 dpbm were compared to those of the DMSO-injected control group. Each mosquito was injected with 3.45 pmole (1.1 ng) or 17.25 pmole (5.5 ng) of MPA. C) DENV titers of mosquitoes injected with either 5 µM BAF, 250 µM MPA, or a cocktail of both compounds were compared to those of the control group. D–E) DENV titers of castanospermine (CAS, 250 mM and 1 mM)- or deoxynojirimycin (DNJ, 250 mM and 1 mM)-injected mosquito midguts at 7 dpbm were compared to those of the control group. F) DENV titers of BAF (5 µM) and MPA (250 µM)-injected mosquito salivary glands at 14 dpbm were reduced compared to those of the DMSO-injected control group. A–F) The red bar indicates a median value of titers. A–B, D–F) Mann-Whitney test. C) Kruskal-Wallis test. ***; p<0.001, **; p<0.01, *; p<0.05. Descriptive statistics for DENV infection assays are presented in supplementary table S2.

To assess the anti-dengue function of BAF, we microinjected 5 µM or 25 µM of BAF into the thorax of 4-day-old female mosquitoes 1 day before allowing them to feed on DENV-infected blood. Microinjection of compounds was used in the first screening as the most effective approach to standardize the timing and quantity of injected compounds. As compared to the untreated controls (n = 42), DENV-fed mosquitoes injected with 5 µM or 25 µM BAF showed a significantly suppressed midgut infection, by 56% (n = 49, p = 0.0002) and 54% (n = 24, p = 0.037), respectively, at 7 days after the infectious blood meal (Fig. 1A). While injection of either 5 µM, 25 µM, 125 µM, or 625 µM BAF did not affect mosquito longevity for up to 16 days (Fig. S1A), injection of 25 µM BAF caused a decreased blood-feeding propensity that resulted in a smaller sample number; therefore we did not use BAF concentrations >25 µM for further DENV inhibition assays. Altogether, these results suggest that BAF has anti-DENV activity in adult mosquitoes.

Thoracic microinjection of mosquitoes with 50 µM or 250 µM of MPA 1 day prior to feeding on virus-infected blood reduced DENV titers of the midgut tissue by 60% in both cases (n = 39, p = 0.0003 and n = 32, p = 0.0003) when compared to the untreated controls (n = 54) at 7 days after feeding on the infectious blood meal (Fig. 1B), indicating that MPA functions as a DENV inhibitor in adult mosquitoes as it does in mammalian and insect cells [13], [17]. Injection of the higher 1.25 mM and 6.25 mM concentrations of MPA resulted in an inconsistent blood feeding propensity, and therefore they could not be used for DENV inhibition assays. Injection of 50 µM, 250 µM, 1.25 mM, or 6.25 mM MPA did not affect the mosquito lifespan for up to 16 days after treatment (Fig. S1B).

We also assessed the ability of two known alpha-glucosidase inhibitors, CAS and DNJ, to influence DENV infection of adult females [14]. Injection of adult female mosquitoes with 250 µM CAS (n = 35, p = 0.0792) or DNJ (n = 40, p = 0.8882), or with 1 mM CAS (n = 38, p = 0.6035) or DNJ (n = 36, p = 0.0039) 1 day prior to feeding on virus-laden blood did not suppress the DENV infection of the midgut tissue when compared to control DMSO-treated mosquitoes (n = 36 or 28) at 7 days after the infectious blood meal (Fig. 1D, E). The data suggest that whereas these two compounds suppress mammalian alpha-glucosidase, they may not function effectively against the mosquito alpha-glucosidase ortholog, or else DENV may utilize alternative enzymes in the mosquito. In an earlier study, we found that RNAi-mediated silencing of an *A. aegypti* alpha-glucosidase ortholog of a *D. melanogaster* gene, previously shown to function as a DENV HF in a fly cell line, did not influence the adult mosquito's susceptibility to DENV infection in the Rock, Sin, or PTri strain of *A. aegypti* [30].

We also investigated the effect of BAF and MPA exposure on salivary gland DENV titers at 14 days post-infection. Injection of 5 µM of BAF or 250 µM of MPA one day prior to feeding on DENV infected blood resulted in reduced viral titers in the salivary glands by 90% (n = 55, p = 0.0005) and 83% (n = 63, p = 0.013), respectively, compared to controls (n = 56). This reduction was greater than that observed in midguts after the same compound treatment (BAF: 56%; MPA: 60%) (Fig. 1F). It is likely that further reduction in salivary gland titers would occur if the compound treatments had taken place at 6–10 days after feeding on DENV infected blood since it would likely result in a more efficient HF inactivation in the infected salivary glands.

### Co-injection of BAF and MPA does not result in synergistic DENV inhibition

We wanted to investigate whether injection of a cocktail of BAF and MPA would result in a synergistic anti-DENV effect that would be greater than that of each compound when used independently. Single treatment with 5 µM BAF (n = 35) or 250 µM MPA (n = 39) reduced DENV titers by 60% and 44%, respectively, when compared to controls (n = 42) (Fig. 1C). Surprisingly, the cocktail containing 5 µM BAF and 250 µM MPA reduced DENV titers by only 44% (n = 41) (Fig. 1C), a level comparable to single treatment with 250 µM MPA, suggesting that there may be some drug interaction between the two compounds or that injection of both compounds resulted in a greater mosquito detoxification activity that diminished the active concentration of one or both compounds in DENV-infected cells.

### Time-dependence of BAF and MPA exposure for anti-DENV action

**Figure 2:**
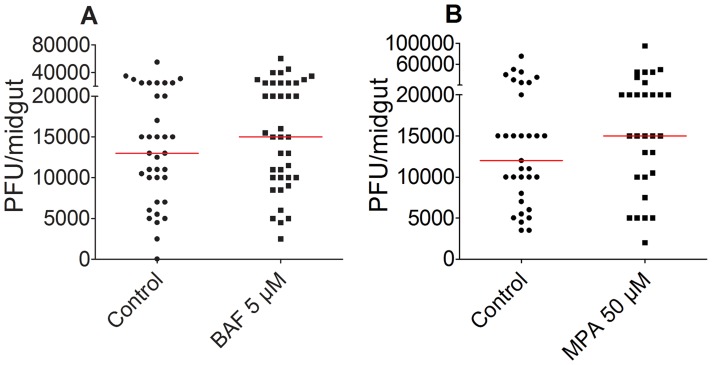
Injection of BAF or MPA after ingestion of virus does not suppress DENV infection. When BAF or MPA was injected 1 day after DENV infection, there was no reduction in DENV titers. P>0.05, Mann-Whitney test. A) Five micromolar BAF was injected into mosquitoes 1 day after DENV infection. B) Fifty micromolar MPA was injected 1 day after DENV infection. Descriptive statistics for DENV infection assays are presented in supplementary table S2.

To determine whether the anti-dengue potency is dependent on whether the compound is injected prior to or after exposure to DENV, we injected mosquitoes with BAF or MPA 1 day after ingestion of a DENV-infected blood meal. Mosquitoes were fed with DENV-infected blood, and the following day, they were injected with 5 µM BAF or 50 µM MPA, or with DMSO as a control. There was no significant difference in DENV titers between the control mosquitoes and those injected with either BAF (n = 39, p = 0.3047) or MPA (n = 33, p = 0.245) (Fig. 2), indicating that the effective antiviral action of these compounds requires application prior or simultaneous with DENV infection.

### Ingestion of BAF and MPA via the blood meal reduces DENV midgut infection

**Figure 3:**
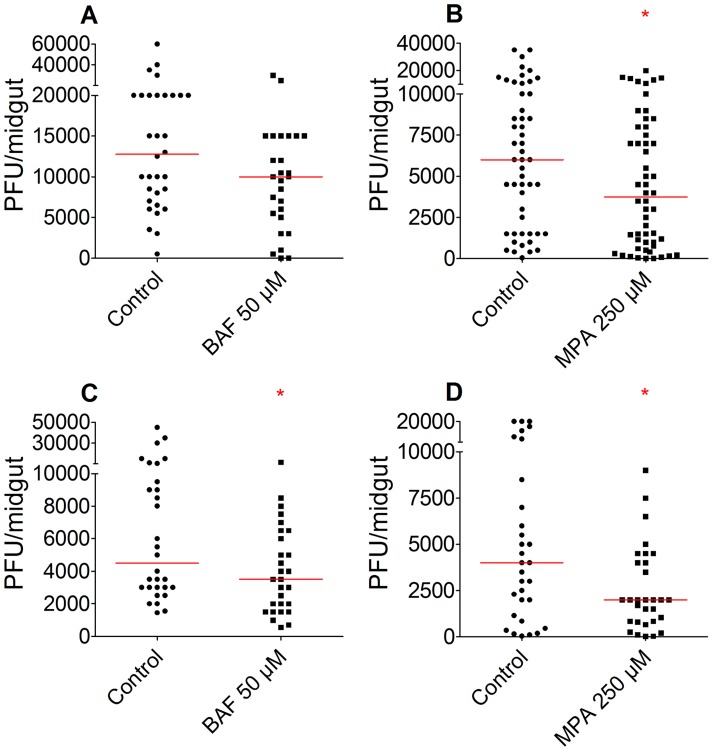
Ingestion of BAF or MPA through the sugar or blood meal results in reduced DENV titers. BAF (50 µM) or MPA (250 µM) was mixed with 10% sucrose (A–B) or an infectious blood (C–D) and fed to mosquitoes. A) DENV titers at 7 dpbm of mosquitoes given BAF in a sugar meal were decreased when compared to the control group, but the difference was not significant (p = 0.0553). B) DENV titers at 7 dpbm of mosquitoes given MPA in a sugar meal were significantly reduced. C) Ingestion of BAF with an infectious blood meal reduced the DENV titers significantly. D) Ingestion of MPA with an infectious blood meal also reduced the titers significantly. (A–D) The red bar indicates a median value of titers. *; p<0.05, Mann-Whitney test. Descriptive statistics for DENV infection assays are presented in supplementary table S2.

Next, we investigated whether BAF and MPA could mediate anti-DENV activity when ingested with either a sugar meal prior to ingestion of a DENV-infected blood meal or together with a DENV-infected blood-meal. For delivery of compounds via a sugar meal, female mosquitoes were maintained on a 10% sucrose solution supplemented with either 50 µM of BAF or 250 µM of MPA or with DMSO (control group) for 2 days prior to, as well as after, feeding on DENV-infected blood. Ingestion of BAF or MPA via the sugar meal decreased DENV titers by 22% (n = 27, p = 0.0553) and 37% (n = 54, p = 0.0351), respectively, when compared to the controls (Fig. 3A, B).

Similarly, ingestion of 50 µM of BAF or 250 µM of MPA, or of DMSO (control group) via the DENV-infected blood also reduced DENV titers by 22% (n = 28, p = 0.0396) and 50% (n = 30, p = 0.0313), respectively, when compared to the controls (Fig. 3C, D).

### The putative BAF and MPA targets, vATPase and IMPDH, serve as mosquito HFs for DENV infection

**Figure 4:**
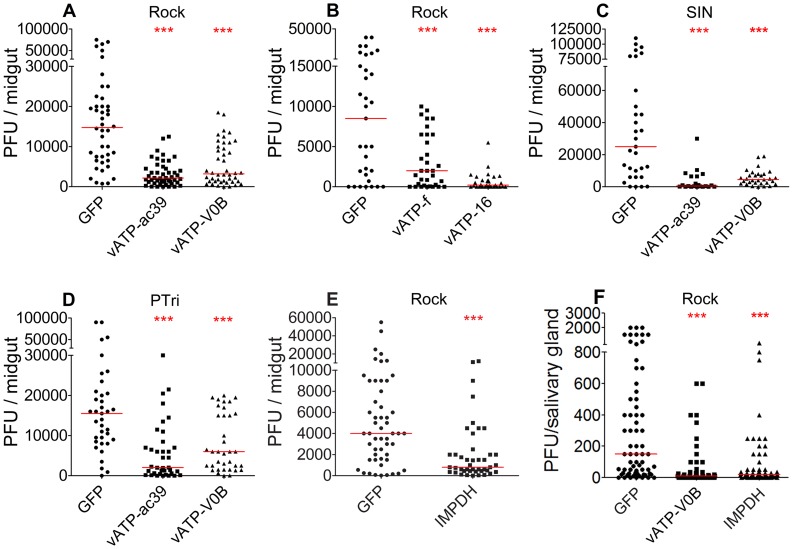
Silencing of vATPase or IMPDH results in suppression of DENV infection. A–B) Silencing of vATPase subunit ac39 (vATP-ac39, AAEL011025), vATPase proteolipid subunit (vATP-V0B, AAEL012113), vATPase subunit f (vATP-f, AAEL002464), and vATPase 16-kDa proteolipid subunit (vATP-16, AAEL000291) reduced DENV titers in the Rock strain, C) in the SIN strain, and D) in the PTri strain. E) Silencing of inosine 5′-monophosphate dehydrogenase (IMPDH, AAEL009273) reduced DENV titers in the Rock strain. F) Silencing of vATPase (vATP-V0B) and IMPDH reduced DENV titers in salivary glands at 14 dpbm. ***; p<0.001, **; p<0.01, Mann-Whitney test. A–F) The red bar indicates a median value of titers. Descriptive statistics for DENV infection assays are presented in supplementary table S2.

BAF has been shown to inhibit viral entry *in vitro* by binding to the multi-subunit vATPase enzyme complex [10], [18], [24], [26], . To date, one vATPase subunit (vATP-g, AAEL012819) has been experimentally confirmed to influence DENV infection in *A. aegypti* mosquitoes [30]. To provide more robust evidence for the activity of vATPase as a DENV HF and to determine whether depletion of other vATPase subunits also negatively affects DENV infection of adult mosquitoes, we performed dsRNA-mediated gene silencing on four additional vATPase subunits. dsRNA-mediated silencing of vATP-ac39, vATP-V0B, vATP-f, and vATP-16 reduced DENV titers by 85%, 78%, 76%, and 98%, respectively, in the Rock (laboratory mosquito) strain (p values<0.01) (Fig. 4A,B). Moreover, silencing of vATP-ac39 and vATP-V0B in two DENV-susceptible field-derived strains, SIN and PTri, also significantly reduced their DENV titers by 98% and 82% in SIN, and by 87% and 61% in PTri, respectively (Fig. 4C, D) [30]. MPA has been suggested to block DENV infection in human cells by inhibiting IMPDH1 and IMPDH2 [34], [35]. There is only one ortholog of IMPDH (AAEL009273) in *A. aegypti* mosquitoes, and its RNAi-mediated silencing resulted in an 80% suppression of DENV infection (p = 0.0001, n = 43) when compared to control GFP dsRNA-injected mosquitoes (n = 53) (Fig. 4E). Our data therefore demonstrate that the targets of BAF and MPA in the *A. aegypti* mosquito are likely to be vATPase and IMPDH, respectively.

**Figure 5:**
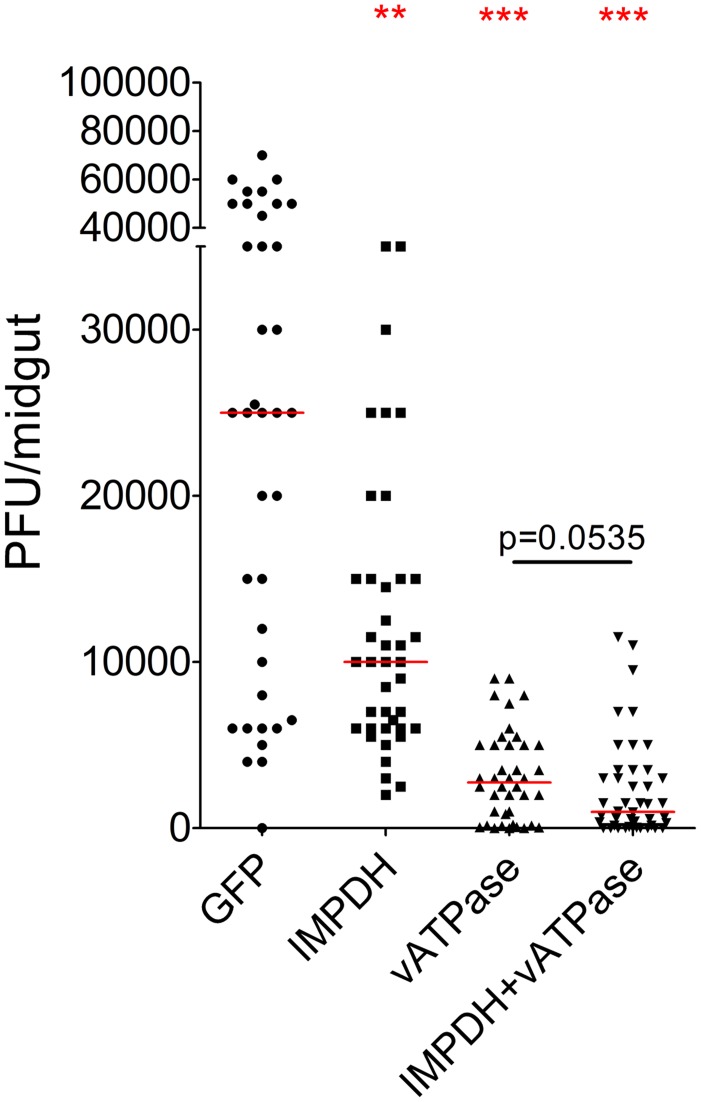
Co-silencing of vATPase (vATP-V0B) and IMPDH. Co-silencing of vATPase (vATP-V0B) and IMPDH reduced DENV titers more than single silencing, although the difference between the co-silenced and vATPase single-silenced mosquito cohorts was not significant (p = 0.0535, Mann-Whitney test). ***; p<0.001, **; p<0.01, Mann-Whitney test. Descriptive statistics for DENV infection assays are presented in supplementary table S2.

Next, we investigated whether co-silencing of vATPase and IMPDH would result in a greater DENV inhibition than when these genes were silenced independently. Simultaneous knockdown of vATP-V0B and IMPDH reduced DENV titers by 96.1%, while silencing of the individual genes decreased DENV titers by 89% (vATP-V0B) and 60% (IMPDH) (Fig. 5). Our data suggest that the synergistic silencing effect of these two HFs on DENV infection is marginal, likely because of a lesser depletion efficiency of each gene transcript and/or protein when co-silenced, or a possible alternative infection route of a small proportion of DENV.

We also measured salivary gland DENV titers after silencing the target genes through injection of dsRNA at 3 days prior to feeding on DENV containing blood. Silencing of vATP-V0B and IMPDH reduced viral titers by 93% (n = 51, p<0.0001) and 87% (n = 63, p<0.0001), respectively. This reduction was greater, although marginal, than that observed in midguts (vATP-V0B: 78%; IMPDH: 80%) (Fig. 4F). It is likely that further reduction in salivary gland titers would occur if the dsRNA treatments had taken place at 6–10 days after feeding on DENV infected blood since it would likely result in HF gene silencing in the infected salivary glands.

### Effects of vATPase and IMPDH silencing on mosquito fitness as a measure of longevity, fecundity, and fertility

**Figure 6:**
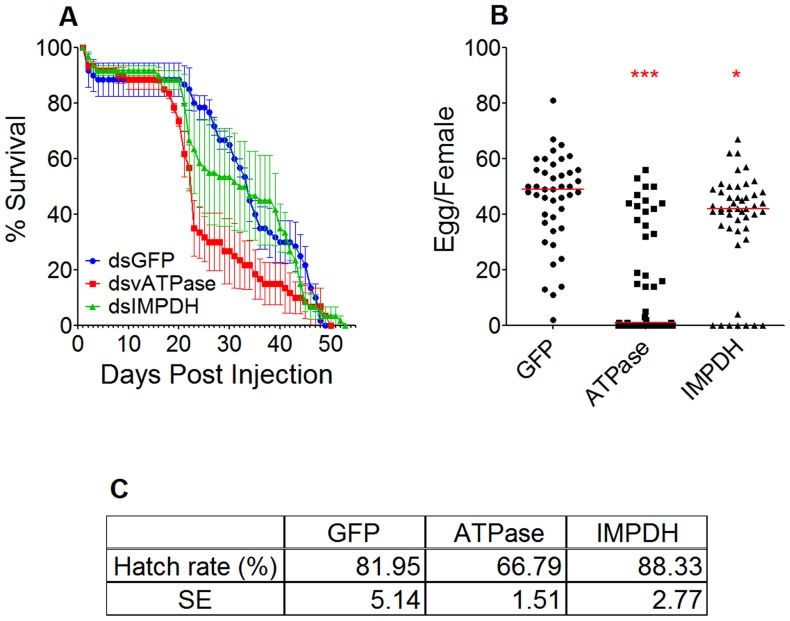
Fitness impact of vATPase and IMPDH silencing. A) Mosquitoes were injected with vATPase dsRNA, IMPDH dsRNA and a longevity study was conducted. Survival was assessed until all mosquitoes had perished. The mean values from three replicates are shown, with the standard error bars. Additionally survival rates were analyzed by Kaplan-Meier survival analysis with Wilcoxon test, and detailed statistical information is available in Table S3. B) Fecundity analysis was conducted after vATPase dsRNA, IMPDH dsRNA was injected. The horizontal bars represent the median value of eggs laid per female. ***; p<0.001, *; p<0.05, Mann-Whitney test. C) Fertility analysis was conducted with the eggs obtained from the fecundity assay. Hatch rates indicate the average percentage of eggs giving rise to first to second instar larvae. Mean values for hatch rates and standard errors (SE) of three biological replicates are indicated.

In order to assess possible fitness effects of HF inactivation we investigated the effects of vATPase and IMPDH silencing on mosquito fitness as a measure of longevity, fecundity, and fertility. Silencing of vATP-V0B significantly affected longevity (2 way ANOVA, p<0.0001), fecundity (98% reduction, p<0.0001) and fertility (19% reduction, p = 0.0475) while silencing of IMPDH showed no or only marginal effects on longevity (p = 0.8124), fecundity (14% reduction, p = 0.0141) and fertility (p = 0.3366) compared to control mosquitoes (Fig. 6).

## Discussion

A variety of arthropod-transmitted virus HFs have been discovered, and some of them have potential as virus transmission-blocking targets [14], [15], [36]–[38]. Here we have investigated the potency of the drugs BAF, MPA, CAS, and DNJ, all of which are known to block DENV infection in mammalian cells, in inhibiting infection of the mosquito vector. vATPase plays a fundamental role in virus membrane fusion as a vacuolar proton pump that acidifies the vacuole [11]. When viruses enter the cell by endocytosis, membrane fusion is necessary to release the virions from the endosome to the cytoplasm, and a key factor in membrane fusion is vacuole acidification [12]. Inhibition of vATPases with BAF, derived from *Streptomyces griseus* [27], has been shown to suppress DENV infection in various mammalian and insect cell lines. BAF suppresses DENV by 80% in the *A. albopictus* cell line C6/36 HT [18] and also inhibits Sindbis virus infection in mammalian BHK cells, but not in *A. albopictus* C7-10 cells [24]. MPA was developed as a transplant rejection preventive drug and is an inhibitor of inosine-5′-monophosphate dehydrogenase (IMPDH) that catalyzes the synthesis of xanthosine monophosphate (XMP) from inosine monophosphate (IMP) [37], [38]. This is a rate-limiting step for the *de novo* synthesis of guanine nucleotides and is required for DNA and RNA synthesis [34]. MPA treatment of various mammalian cells has been shown to suppress DENV infection [13], [17]. Treatment with MPA also inhibits Sindbis virus infection of the *A. albopictus* cell lines, LTC 7 and Ama 18, suggesting MPA can act as an antiviral compound across taxa [39]. In our present study, Injection of either 50 or 250 µM MPA suppressed DENV infection in adult mosquitoes, and there was no difference of the reduction produced by the two concentrations, suggesting that IMPDH inhibition by MPA is already saturated at 50 µM.

CAS and DNJ are inhibitors of alpha-glucosidases that function as DENV HFs in mammals [14], [15]. An alpha-glucosidase has also been identified as a possible DENV HF in the *D. melanogaster* cell line S2 [10], and its ortholog is induced in *A. aegypti* midgut during DENV-2 infection [40], suggesting that it may represent a DENV HF in the mosquito. However, our results showed that none of the alpha-glucosidase inhibitors suppressed DENV infection of adult mosquitoes (Fig. 1D, E), nor did RNAi-mediated silencing of the alpha-glucosidase gene (AAEL010599 and AAEL015337), an ortholog of a *D. melanogaster* DENV HF, result in altered susceptibility to the virus [30]. These results suggest that DENV can utilize alternative enzymes in *A. aegypti* that are resistant to DNJ and CAS treatment. However, we cannot exclude the possibility that another *A. aegypti* alpha-glucosidase that is resistant to CAS and DNJ treatment serves as a DENV HF.

Effective antiviral action of BAF and MPA required administration prior, or simultaneously, to virus exposure, whereas compound injection into mosquitoes at 24 h after feeding on DENV infected blood did not result in a significant DENV suppression in the midgut tissue. BAF inhibits virus entry and MPA inhibits replication, and at 24 h after ingestion of the virus-laden blood, a sufficient number of viruses may already have succeeded in entering the cells and replicating, thereby exceeding the threshold above which the drug's antiviral effect is ineffective. However, it is likely that BAF and MPA treatment after feeding on DENV infected blood could result in virus suppression in the salivary glands. We did not inject mosquitoes with the compounds at the time of infected blood ingestion to avoid interfering with the feeding. However, ingestion of the compounds together with DENV via a blood meal resulted in suppression of DENV. Thus, inhibition of HFs with BAF and MPA must occur prior to infection, or during the early stage of infection, in order to block viral infection of the mosquito.

Despite the fact that BAF (an inhibitor of virus cell entry) and MPA (an inhibitor of viral RNA synthesis) inhibit the virus at two independent stages of infection, no synergistic antiviral effect was observed when the compounds were administered simultaneously. However, in contrast to the co-injection of the two compounds, RNAi-mediated co-silencing of the putative drug target genes vATPase and IMPDH did result in a marginally greater antiviral effect when compared to silencing each gene individually (the p-value of the difference in virus titer between the co-silenced and single-silenced mosquito cohorts was just above the significant level: p = 0.0535). This discrepancy is a likely result of a drug interaction between BAF and MPA that negatively affects the activity of one of the two, or of both drugs when administered together. Alternatively, the large amount of xenobiotics in co-injected mosquitoes may have augmented the insect's detoxification system, thereby neutralizing the action of any of the administered compounds.

Although injection may be the most efficient way to deliver these compounds, it is not a natural route that could be applied to mosquitoes in nature; however, in nature, one of the following three routes would be possible: ingestion through the nectar, through blood feeding, or through surface exposure as in the case of insecticide treatment. Ingestion of toxic mosquitocidal substances through artificial nectar feeding has been deployed for mosquito control [41], [42], but ingestion of mosquitocidal or transmission-blocking substances through blood feeding has only been addressed by one previous study that investigated the effect on the mosquito but not virus infection [43]. Thus, we investigated the possibility of compound delivery through blood feeding. This route of mosquito exposure to compounds would simulate a situation when mosquitoes would feed on a dengue infected individual being treated with therapeutic and transmission-blocking medication. Our data show that this would have the potential to suppress DENV replication and thus limit the likelihood of DENV transmission.

The greater antiviral efficacy of the injected versus ingested compounds is most likely due to the fact that the injection brings the compound directly into the hemolymph, where it can be absorbed by the basal side of the midgut epithelial cells. In contrast, in order to exert antiviral activity through HF inhibition, compounds ingested via the blood would have to traverse the chitinous peritrophic matrix and be absorbed by the lumenar side of the midgut epithelial cells, in addition to persisting in the blood meal [44]–[46]. Furthermore, a large proportion of the ingested compounds may be excreted through diuresis rather than being absorbed by the midgut epithelium [47]. Administration through the sugar meal will most likely result in a lower overall uptake of a compound, since the amount of ingested sugar is smaller than that of blood and it occurs over a longer period that might not allow the active concentration of the compound to reach potent levels.

By silencing the respective genes in adult mosquitoes prior to DENV infection, we confirmed that the predicted targets of BAF and MPA are associated with DENV replication. BAF inhibits the vATPase by binding to a proteolipid subunit of the vATPase V0 domain [27], [48]. vATPases are multisubunit enzymes with two domains. In yeast, the peripheral catalytic V1 domain has eight subunits and the integral membrane V0 domain has six subunits [Reviewed in 49]. We have previously confirmed that vATPase subunit G (AAEL012819) is a DENV HF [30]. In the current study, we have tested four additional subunits of the vATPase, including two of the proteolipid subunits (AAEL012113, AAEL000291) of V0 that are known to be inhibited by BAF. vATP-f is an ortholog of the vATPase V1 domain subunit in yeast, and the other tested genes are also orthologs of vATPase V0 domain subunits. The *A. aegypti* orthologs of the V0 domains, AAEL011025, AAEL012113, and AAEL000291, have been shown to be up-regulated in the DENV-susceptible Moyo-S strain in response to DENV infection, further indicating that vATPases play important roles in viral infection [50]. Our RNAi-mediated gene silencing of all four tested vATPase subunits resulted a significant inhibition of DENV, suggesting that the function of vATPase enzyme as a whole complex is required for efficient DENV infection in *A. aegypti*. This gene silencing-mediated infection phenotype was consistent across laboratory-adapted and field-derived mosquito strains, emphasizing a crucial role for this enzyme complex in DENV infection of adult mosquitoes. Thus, vATPase is a highly potent candidate target for DENV HF inhibitors. Effects on IMPDH is the target enzyme of MPA and a known virus HF involved in viral replication [51], [52], and its depletion through RNAi also reduced DENV titers, suggesting that it represents a druggable DENV HF in adult *A. aegypti*.

Silencing of the vATPase subunit negatively affected mosquito fitness; a decreased mosquito lifespan was observed from 17 days post injection and onwards. This observation is consistent with findings from earlier studies on yeast [53], [54], *Neurospora* [55], mice [56] and insects including *Drosophila melanogaster*, *Tribolium castaneum*, *Acyrthosiphon pisum*, *Manduca sexta*, *Peregrinus maidis*, *Diabrotica virgifera*, *Bemisia tabaci* and *Bactrocera dorsalis* [57]–[61]. Defective vATPase in these organisms resulted in greater mortality during various life stages including embryonic, larval and adult stages, indicating that vATPase function is critical for homeostasis. Impaired function of vATPase in adult female mosquitoes also impeded oviposition. Following vATPase subunit silencing, fecundity, as measured by the median number of eggs produced, was reduced by 98%. Reduced fecundity as a result of vATPase silencing has been reported in horn flies and corn planthoppers [59], [62]. In corn planthoppers, silencing of vATPase caused immature oocyte formation [59]. Our data show that chemical or dsRNA-mediated inactivation of vATPase has the potential to result in mosquito population suppression. Earlier studies have also proposed that dsRNAs targeting of vATPases could be used to target insect pests [58]–[60]. The synergistic effects of vATPase inactivation on mosquito fitness and DENV infection potentiates the targeting of this HF for transmission blocking. However, in the case of chemical vATPase inactivation the non-specific effects due to the high degree of vATPases conservation across species must be considered.

In summary, we show here for the first time that several vATPase subunits and IMPDH represent potent HFs in adult female *A. aegypti* and that they can be targeted by drugs to inhibit DENV infection and potentially transmission. The development of dengue control strategies based on the chemical or RNAi-mediated inhibition [63]–[65] of virus HFs has several advantages, since it does not have the ecological impact of insecticides that indiscriminately kill insects, and it is not subject to the virus's ability to develop resistance. HF-targeted compounds or dsRNAs can be delivered through artificial nectar [66]. Alternatively, they can be engineered, or administered through an appropriate medium, for effective diffusion through the insect's cuticle and then be applied as sprays or impregnated into bed nets, as in the case of insecticides. Many DENV HFs, for example vATPase and IMPDH, are shared between the mosquito and human hosts and therefore open the way for the discovery of drugs that have both therapeutic (i.e., blocking the virus in the human host) and transmission-blocking (i.e., blocking the virus in the mosquito vector) activity, thereby maximizing the disease-control potential. Although BAF and MPA exert anti-viral activity in the mosquito, they are likely not optimal, from a drug efficacy or economic standpoint, for dengue control. However, a knowledge of vATPase and IMPDH as ubiquitous HFs in humans and mosquitoes, along with the structural attributes of BAF and MPA and other known anti-dengue compound point to the possibility of developing effective chemicals that could exert both therapeutic and transmission blocking activity for dengue control.

## Supporting Information

Figure S1 **Survival curves of mosquitoes after injection with various concentrations of BAF or MPA.** The survival percentage is given for 30 mosquitoes in each group. The significance of the effect of the compounds on the mortality of mosquitoes, when compared to DMSO-injected controls, was determined by Kaplan–Meier survival analysis using GraphPad Prism (Prism 5.05; GraphPad Software, Inc.), and p-values were calculated with the Wilcoxon test [67]. A) Various concentrations of BAF (5, 25, 125 and 625 µM) were injected into mosquitoes, and mortality was observed for 16 days (p>0.05). B) Various concentrations of MPA (0.005, 0.025, 1.25 and 6.25 mM) were injected into mosquitoes, and mortality was observed for 16 days (p>0.05).
(TIF)

Figure S2 **Silencing efficiency of target genes.** vATPase subunits were tested in the Rockefeller (Rock), Singapore (SIN) and Puerto Triunfo strains (PTri). IMPDH was tested in the Rock strain.
(TIF)

Table S1 PCR primer sequences.
(XLS)

Table S2 Descriptive statistics for DENV infection assays.
(XLS)

Table S3 Survival analysis of mosquitoes after silencing.
(XLS)
