# Supplementary figures and images for: Suppressing Dengue-2 Infection by Chemical Inhibition of Aedes aegypti Host Factors
Source: PLoS Negl Trop Dis. 2014 Aug 7;8(8):e3084. doi: 10.1371/journal.pntd.0003084 (PMC4125141; doi:10.1371/journal.pntd.0003084)

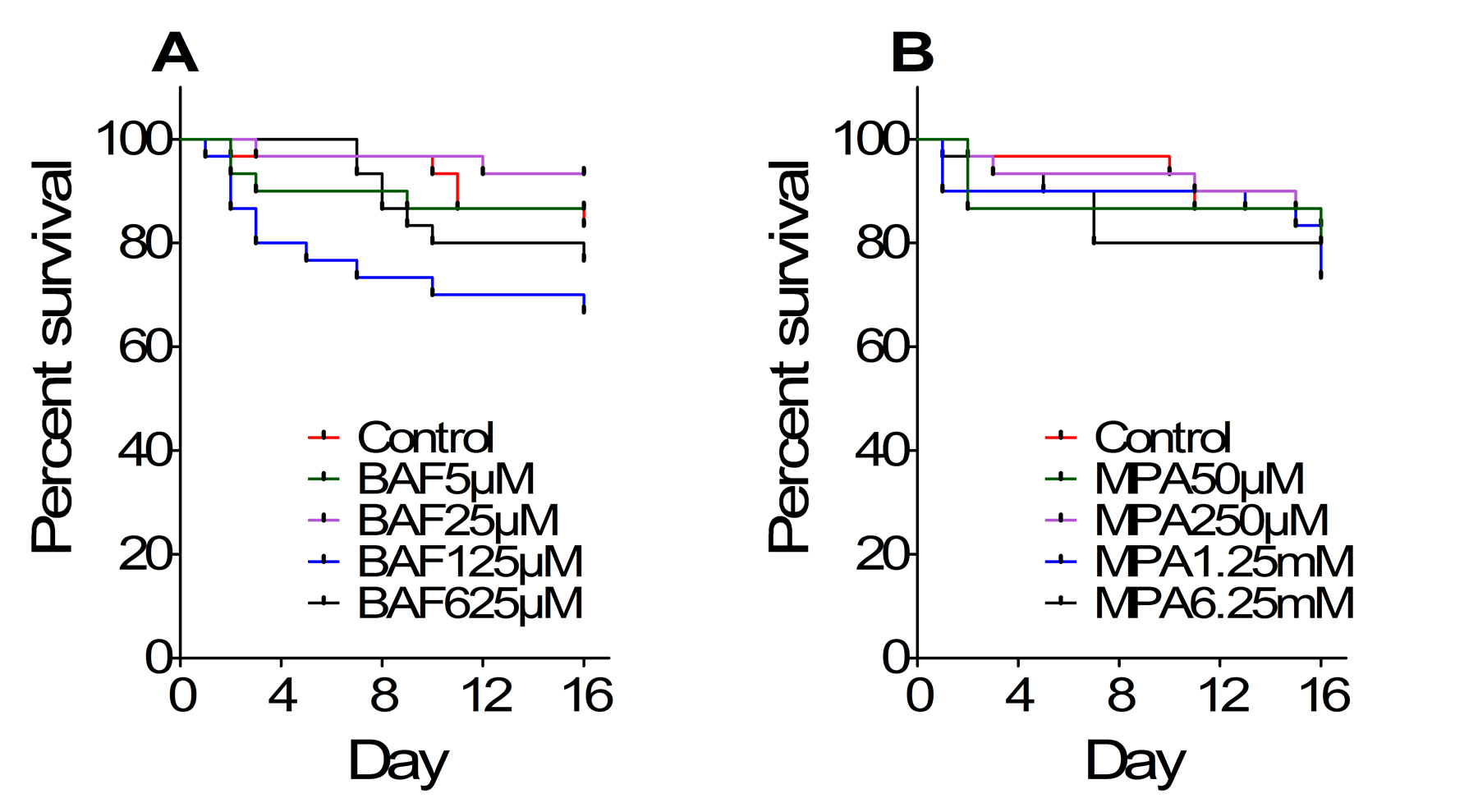

Supplement: Figure S1 — Survival curves of mosquitoes after injection with various concentrations of BAF or MPA. The survival percentage is given for 30 mosquitoes in each group. The significance of the effect of the compounds on the mortality of mosquitoes, when compared to DMSO-injected controls, was determined by Kaplan–Meier survival analysis using GraphPad Prism (Prism 5.05; GraphPad Software, Inc.), and p-values were calculated with the Wilcoxon test [67]. A) Various concentrations of BAF (5, 25, 125 and 625 µM) were injected into mosquitoes, and mortality was observed for 16 days (p>0.05). B) Various concentrations of MPA (0.005, 0.025, 1.25 and 6.25 mM) were injected into mosquitoes, and mortality was observed for 16 days (p>0.05). (TIF) [file pntd.0003084.s001.tif]

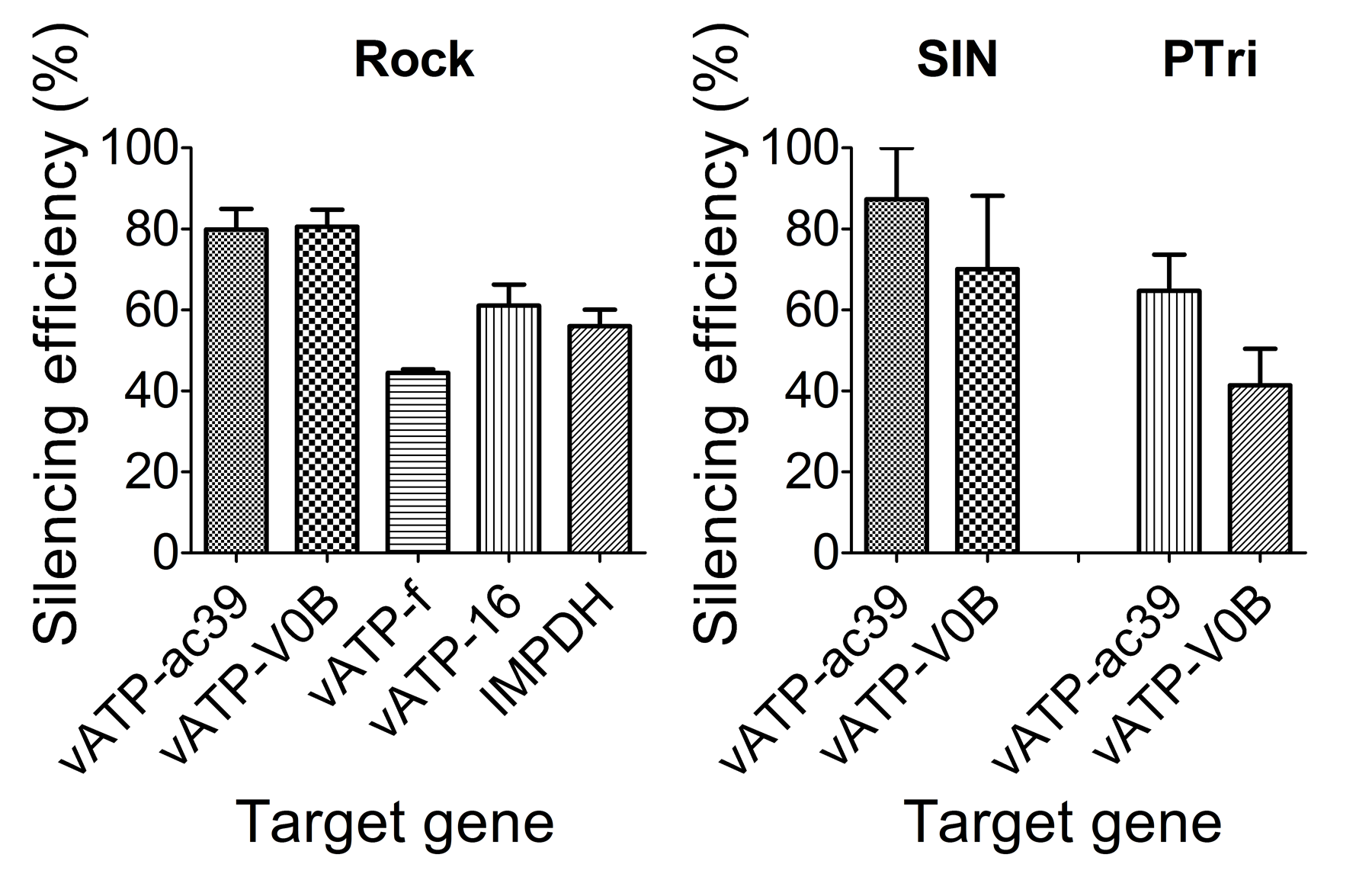

Supplement: Figure S2 — Silencing efficiency of target genes. vATPase subunits were tested in the Rockefeller (Rock), Singapore (SIN) and Puerto Triunfo strains (PTri). IMPDH was tested in the Rock strain. (TIF) [file pntd.0003084.s002.tif]
